# Characterisation of resistance mechanisms developed by basal cell carcinoma cells in response to repeated cycles of Photodynamic Therapy

**DOI:** 10.1038/s41598-019-41313-y

**Published:** 2019-03-18

**Authors:** Silvia Rocio Lucena, Alicia Zamarrón, Elisa Carrasco, Miguel Angel Marigil, Marta Mascaraque, Montserrat Fernández-Guarino, Yolanda Gilaberte, Salvador González, Angeles Juarranz

**Affiliations:** 1 Biology Department, Faculty of Sciences, Autonomous University of Madrid, Madrid, Spain; 2 Molecular Biology Department, Faculty of Sciences, Autonomous University of Madrid, Madrid, Spain; 3 Pathology Service, Hospital San Jorge, Huesca, Spain; 4 Dermatology Service, Hospital Ramón y Cajal, Madrid, Spain; 5 Dermatology Service, Hospital Miguel Servet, Zaragoza, Spain; 6 Medicine and Medical Specialties Department, Alcalá de Henares University, Madrid, Spain; 7 Instituto Ramón y Cajal de Investigaciones Sanitarias, IRYCIS, Madrid, Spain

## Abstract

Photodynamic Therapy (PDT) with methyl-aminolevulinate acid (MAL-PDT) is being used for the treatment of Basal cell carcinoma (BCC), but recurrences have been reported. In this work, we have evaluated resistance mechanisms to MAL-PDT developed by three BCC cell lines (ASZ, BSZ and CSZ), derived from mice on a *ptch*+/− background and with or without *p53* expression, subjected to 10 cycles of PDT (10^th^G). The resistant populations showed mesenchymal-like structure and diminished proliferative capacity and size compared to the parental (P) cells. The resistance was dependent on the production of the endogenous photosensitiser protoporphyrin IX in the CSZ cell line and on its cellular localisation in ASZ and BSZ cells. Moreover, resistant cells expressing the *p53* gene presented lower proliferation rate and increased expression levels of N-cadherin and Gsk3β (a component of the Wnt/β-catenin pathway) than P cells. In contrast, 10^th^G cells lacking the *p53* gene showed lower levels of expression of Gsk3β in the cytoplasm and of E-cadherin and β-catenin in the membrane. In addition, resistant cells presented higher tumorigenic ability in immunosuppressed mice. Altogether, these results shed light on resistance mechanisms of BCC to PDT and may help to improve the use of this therapeutic approach.

## Introduction

Basal cell carcinoma (BCC) is the most prevalent skin cancer worldwide^1^. BCC can be highly mutilating, destroying the surrounding tissue, and its recurrence rate is relatively high, reappearing on a 10–20% of the patients 5 years after treatment^2^. BCC is a complex malignancy that can appear spontaneously or be due to predisposing genetic syndromes, like Gorlin-Goltz or Xeroderma Pigmentosum. Independently from its origin, in most cases, Hedgehog (Hh) signalling pathway is altered^3,4^ and *P53* is mutated in the 50% of human BCCs^5^. In addition, mutations on genes involved in the Hh pathway have been described in sporadic BCCs or in those induced by carcinogens, such as ultraviolet (UV) irradiation. Between 50–70% of BCCs showed inactivating mutations in PTCH-1, the receptor of Hh^6^.

There are several therapies approved by FDA for the treatment of BCCs. The most commonly used is surgery. However, as BCC usually appears on the face, neck or extremities, non-invasive therapies such as topical Imiquimod or Photodynamic Therapy (PDT)^7,8^ have been developed and approved by regulatory agencies. PDT consists in the administration of a photosensitiser (PS), which is then excited by light of appropriate wavelength in the presence of oxygen. The reaction causes cell death through the production of reactive oxygen species (ROS). One of the compounds approved for its use in oncologic dermatology is MAL (Methyl aminolevulinate), a precursor of the endogenous PS protoporphyrin IX (PpIX). The PpIX is an intermediate of the heme biosynthesis route that accumulates preferentially in cancer cells^9–11^.

Despite all PDT advantages, recurrence may occur after the treatment, as it happens with many other oncological therapies. Resistance to cancer treatments is thought to be the main cause for treatment failure and relapse. Thus, the identification of the mechanisms involved in resistance constitutes an important objective for the development of new strategies to overcome it. These resistance mechanisms have been scarcely studied for PDT, especially in BCC. Some of the intracellular PDT resistance mechanisms identified are similar for other treatments, and are associated with: changes in expression of proteins related to cell death, like P53; constitutive activation of Wnt/β-catenin pathway; epithelial to mesenchymal transition (EMT); or presence of cancer stem cells^12–14^.

We hypothesized that resistance occurs in three BCC murine cell lines (ASZ, BSZ and CSZ), obtained from tumours induced in heterozygous mice for *ptch* (*ptch*^+/−^) and with or without the gene *p53*, after 10 cycles of MAL-PDT (10^th^G). Therefore, our principal objective was to identify factors implicated in this resistance process. The results obtained revealed that resistant cells (10^th^G) showed different phenotype compared to their corresponding parental cells (P), depending on the presence of *p53* or on their different origin. On a step forward, when resistant and parental cells were inoculated into immunosuppressed mice *in vivo*, we observed a more aggressive behaviour of the cells isolated from tumours generated from 10^th^G cells (10^th^G T) than those induced by parental cells (P T).

## Results

### Generation of cell lines resistant to MAL-PDT

**Figure 1:**
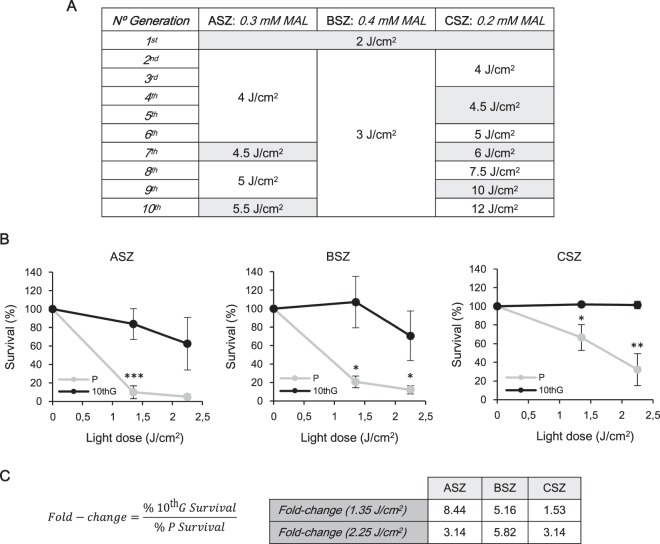
Generation of PDT resistance. (**A**) Red light doses and MAL concentrations used to obtain resistant populations to 10 cycles of PDT (10^th^G) are summarized in the table. (**B**) PDT resistance of 10^th^G respect to their corresponding P cells was analysed by MTT assay. For each cell line the MAL concentration used were: 0.3 mM ASZ; 0.4 mM BSZ; 0.2 mM CSZ. Error bars represent SD (n = 4). (**C**) Fold-change index was calculated with the formula shown. (*P ≤ 0.05; **P ≤ 0.01; ***P ≤ 0.001).

To generate BCC murine cell lines highly resistant to PDT, ASZ, BSZ and CSZ parental (P) cell lines were subjected to 10 cycles of PDT, giving rise to each 10^th^ generation cells (10^th^G), respectively. The procedure followed was based on those previously published for isolation of PDT resistant mouse or human cancer cells^15–18^. To this end, cell lines were subjected to increasing doses of red light, maintaining fixed the concentrations of MAL (ASZ 0.3 mM, BSZ 0.4 mM and CSZ 0.2 mM) along the consecutive cycles (Fig. 1A). Treatment conditions that caused survival rates of 5–15% were chosen.

The resistance of 10^th^G populations was validated comparing the response, in terms of cell survival, to PDT using the MAL concentrations indicated above and two doses of light. All three 10^th^G cells were significantly more resistant than their respective P and fold-change indexes (ratio between the % of survival of each 10^th^G and P cell type after PDT) were greater than 1 in all cases (Fig. 1B).

### Cell morphology, size and complexity

**Figure 2:**
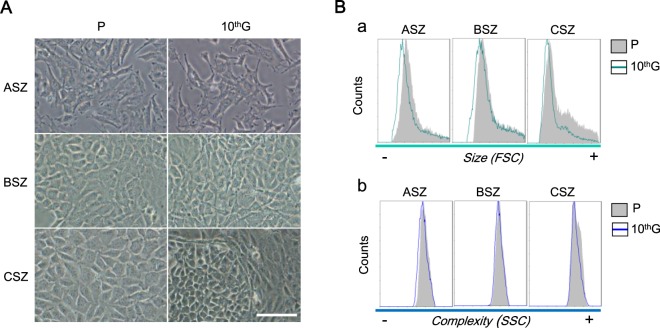
Cell morphology, size and complexity of P and 10^th^G populations. (**A**) Images of cells obtained under phase contrast microscopy. The presence of polygonal cells is indicated with an asterisk and lengthened cells with an arrow. Scale bar: 60 µm. (**B**) A representative experiment (three repetitions) of flow cytometry is shown, in which (a) forward scatter represents size and (b) side scatter cell complexity.

The general morphology of the cells was analysed by phase contrast microscopy. ASZ P cells tended to grow separated from each other, while BSZ P and CSZ P grew with a more compact pattern, forming well-defined colonies of polygonal cells. ASZ 10^th^G did not show remarkable morphological differences compared with P, but a higher presence of elongated cells did appear in cultures. BSZ 10^th^G cells presented a more fibroblastic shape than their corresponding P. The most relevant change was noticed in CSZ 10^th^G cultures, in which two different morphologies could be recognized: one similar to P (polygonal cells in colonies) and the other one with a fibroblastic shape, the latter surrounding the former (Fig. 2A).

Cell size analyses revealed that 10^th^G cells were smaller than their corresponding P, particularly for CSZ, whose 10^th^G population showed a remarkable decrease of larger cells. Cell complexity was similar in ASZ and BSZ P compared to their respective 10^th^G populations, whereas CSZ 10^th^G culture was more heterogeneous than that of CSZ P (Fig. 2B).

### Proliferation capacity, adhesion efficiency and cell cycle

**Figure 3:**
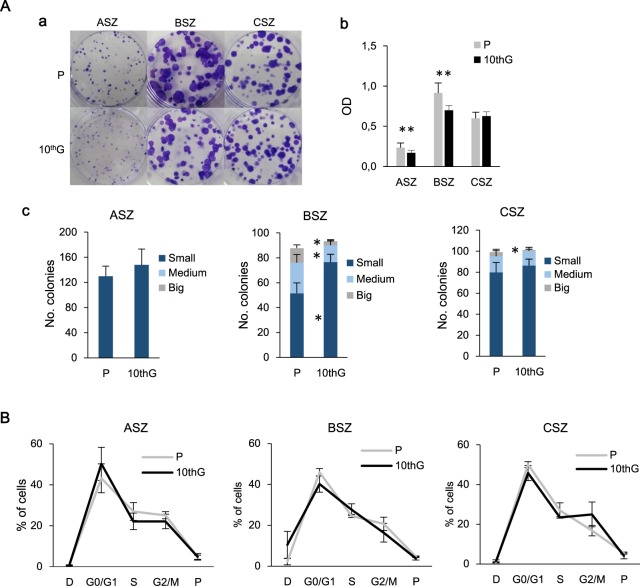
Proliferation capacity and cell cycle analysis. (**A**) For the clonogenic assay, 125 cells/mL were seeded in each plate and 7 days later, colonies were stained with 0.2% crystal violet; it is shown a representative experiment (a); (b) crystal violet was dissolved in SDS and optic density at 570 nm was measured (**P ≤ 0.01) (c) colonies were counted and classified in relation to their diameter (<1.5 mm: small; >1.5 mm, <2.5 mm: medium; >2.5 mm: big) (*P ≤ 0.05). Values are represented as mean ± SD (n = 3); (**B**) It is represented the mean value ± SD (n = 3) of the percentage of parental (P) and resistant (10^th^G) cells in each cell cycle stage obtained by flow cytometry.

By using the clonogenic assay, we evaluated both the ability to form colonies (each colony is formed by >50 cells) and the proliferative capacity of each cell population, by quantifying the number and the size of the colonies formed, respectively. ASZ cells formed a higher number of colonies although they were small in size, while BSZ and CSZ gave rise to a lower number of colonies of different sizes. Comparing P and 10^th^G populations, no differences in the number of colonies were observed. Regarding the size, BSZ P and CSZ P formed significantly higher number of large colonies than 10^th^G. Crystal violet staining absorbance indicated a higher cellular density of ASZ and BSZ P in relation to their 10^th^G (Fig. 3A). To conclude, ASZ (P and 10^th^G) presented higher adhesion capacity than BSZ and CSZ cells since they formed a higher number of colonies. Conversely, BSZ and CSZ cells formed larger colonies, which indicate that they are more proliferative.

No differences were found in the distribution of the three cell lines among the different phases of the cell cycle, with approximately 50% of cells in G0/G1 phase, 25% in S phase and 20–25% in G2/M. No differences were found between P and 10^th^G for any of the cell lines (Fig. 3B).

### Subcellular localisation and production of Protoporphyrin IX

**Figure 4:**
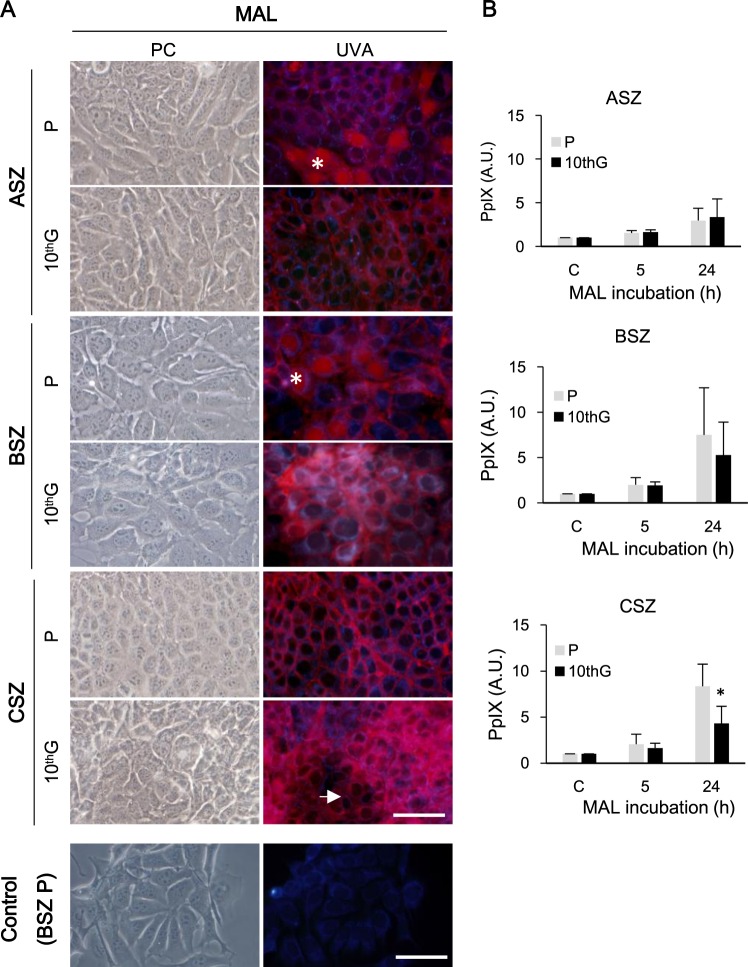
Localisation and production of protoporphyrin IX (PpIX). (**A**) PpIX localisation was evaluated after 24 h of incubation with its precursor MAL (0.3 mM ASZ; 0.4 mM BSZ; 0.2 mM CSZ) by fluorescence microscopy under UVA excitation light, checking cell integrity by phase contrast microscopy (PC). Asterisks indicate nuclear localisation and the arrow points to spindled-shaped cells with lower PpIX signal. Representative cells (BSZ P) without incubation with MAL (Control) are included as an autofluorescence control under UVA excitation light. Scale bar: 60 µm. (**B**) PpIX production was evaluated by flow cytometry after 5 or 24 h of incubation with MAL (*P ≤ 0.05).

To determine PpIX localisation, P and 10^th^G cells were observed by fluorescence microscopy after 24 h of incubation with appropriate MAL concentrations (ASZ 0.3 mM, BSZ 0.4 mM and CSZ 0.2 mM) under UVA excitation light (Fig. 4A). PpIX was localised in the plasma membrane in all populations; and a very low fluorescence was also detected in the cytoplasm. Besides, nuclear fluorescence signal was detected in ASZ P and BSZ P and scarcely in ASZ 10^th^G. In CSZ 10^th^G, the fluorescent signal was higher in the polygonal than in the spindled cells. In addition, a bluish autofluorescence in the cytoplasm and particularly in mitochondria was observed in control cells without MAL (Fig. 4A; Suppl. Fig. 1A).

Since the amount of intracellular PpIX might affect the phototoxicity, we next examined the PpIX content by flow cytometry (λexc = 625 nm, after incubation with the fixed MAL concentrations for 5 and 24 h) (Fig. 4B). All populations showed higher intracellular PpIX content after 24 h than after 5 h of MAL incubation. There were no significant differences in PpIX production either at 5 or 24 h of incubation between P and 10^th^G, except for CSZ at 24 h, in which 10^th^G produced less PpIX than P.

To evaluate the potential relation between PpIX production and cell death we used the AO-EB assay that allowed distinguishing between viable (fluorescing in green) and dead (fluorescing in orange-red) cells after PDT (Suppl. Fig. 1B). After incubation for 5 h with MAL followed by irradiation, P cultures presented a higher percentage of cell death than 10^th^G, confirming the MTT results. However, in CSZ, when MAL incubation was for 24 h, time for which P cells presented a high PpIX production, differences between P and 10^th^G cell death were higher than at 5 h, confirming that a higher PpIX production is linked to a higher PDT-MAL effectiveness.

### *In vivo* studies: tumorigenic capacity of BCC lines

**Figure 5:**
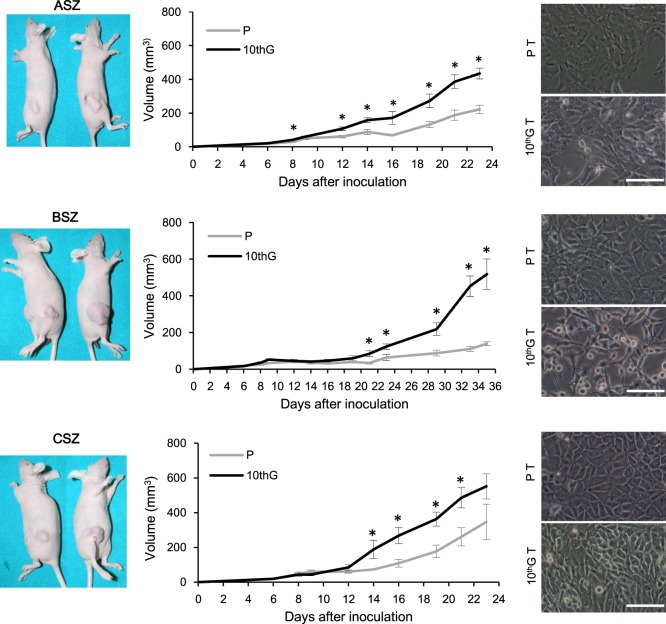
Tumorigenic capacity in a murine model. Cells (1.5 × 10^6^ in 50 µL of PBS and 50 µL of Matrigel) were inoculated subcutaneously in mice, parental (P) in the left and PDT-resistant (10^th^G) cells in the right flank. The mean and SE of tumour volume are presented in the graphs. Human end point of the experiment was determined by a maximum tumour size of 400 mm^3^ (*P ≤ 0.05). Cell morphology is shown in phase contrast microscope images of cells isolated, by explant from the murine tumours. Scale bar: 60 µm.

The tumorigenic capacity of P and 10^th^G populations was evaluated in immunosuppressed mice. After subcutaneous injection into mice, all populations generated tumours. Tumours induced by 10^th^G were bigger than those caused by P cells (*P* ≤ 0.05). ASZ and CSZ lines grew quickly, reaching tumour maximum size of 500 mm^3^ in 23 days, while BSZ was the slowest, needing to be monitored for 35 days. ASZ presented differences in tumour size between P and 10^th^G since day 8 after the inoculation, BSZ since day 21 and CSZ since day 14 (Fig. 5). ASZ generated the smallest tumours; BSZ tumours showed the highest size difference between P and 10^th^G; and CSZ (P and 10^th^G) generated the largest tumours. Tumours formed by ASZ P and 10^th^G cells presented two patterns: the first one was mixed, combining fibroblast-like with fibrosarcoma and osteosarcoma-like cell morphologies, and the second one was composed by fibroblast-like cells only. Tumours induced by BSZ and CSZ P cells exhibited similar characteristics. However, BSZ 10^th^G tumours were composed by fibroblast-like cells and some hemangiopericytoma-like areas (with abundant vascularization). Tumours from CSZ 10^th^G cells showed the three aforementioned morphologies: fibroblast-like, osteosarcoma-like and hemangiopericytoma-like. In general, the tumour progression from both P and 10^th^G cells of the three lines was accompanied by invasion of the muscle and the adipose tissue without inflammation (Suppl. Fig. 2). The areas of calcified osteoid, characteristic of osteogenic differentiation, were stained by alizarin red and were observed only in tumours generated by BSZ P and CSZ (P and 10^th^G) (Suppl. Fig. 2).

In order to characterise the cells composing the tumours, we obtained two new cell populations related to each line (P T and 10^th^G T) by explant culture of the tumours, and studied the cell morphology by phase contrast microscopy (Fig. 5). Both ASZ populations showed a more evident fibroblastoid morphology than the original ones. The most relevant change was observed for BSZ cells, whose 10^th^G T population showed a clear spiky shape *vs*. the polygonal morphology of 10^th^G cells. No evident morphological changes in CSZ T were observed.

### Genetic validation

We next evaluated the expression of *p53* and of *ptch* and their protein products by RT-PCR and Western blot (WB), respectively. The results obtained (Suppl. Fig. 3) confirmed some of those reported by So *et al*.^19^. As expected, no *p53* expression was detected for BSZ and CSZ, as both copies of the *p53* alleles had been ‘floxed’ out. Only cells derived from the ASZ cell line (ASZ 10^th^G, P T and 10^th^G T) expressed the gene *p53* as their corresponding P cells did. We have also checked the status of *p53* in ASZ at the exons 5 and 8, which correspond to certain ‘hot-spots’ in the *p53* gene where mutations are commonly found^20^. In particular, we have found changes in exon 5 at codons 149 (CCA to CTA) and 176 (CAT to CT/AT), but not at codon 137 (ACG) neither at the exon 8, codon 275 (CCT) as previously described^20^. At the protein level, the evaluation by WB validated the expression of p53 in all ASZ populations and no differences were observed between P and 10^th^G. However, its expression was significantly higher in 10^th^G T than in P T (Suppl. Fig. 3A). The expression of *ptch* was also studied by RT-PCR, confirming that 10^th^G, P T and 10^th^G T cell populations had only the mutated form of the gene and they were lacking the wild type one as their corresponding P^19^. The murine fibroblast cell line 3T3 was used as a positive control of the wild type sequence (Suppl. Fig. 3B).

### Evaluation of proteins implicated in tumour progression

It is well documented that during tumour progression, cells can lose their epithelial characteristics and acquire a mesenchymal phenotype that confers invasive properties in an EMT process. In this context, we have evaluated the expression and localisation of some markers related with this process, including: E-cadherin, N-cadherin, vimentin and components of the Wnt/β-catenin pathway.

**Figure 6:**
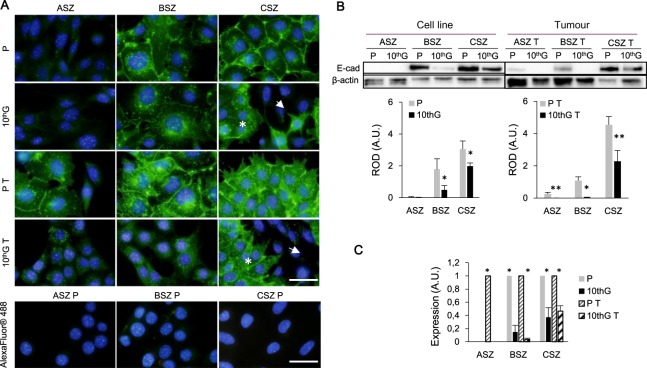
Localisation and expression of E-cadherin. (**A**) Immunofluorescence images of E-cadherin expression in green and DNA staining (DAPI) in blue. A secondary antibody control (AlexaFluor 488) for P cells is included. In CSZ, polygonal population is indicated with an asterisk and spindled cells with and an arrow. Scale bar: 40 µm. (**B**) A representative experiment of E-cadherin expression by Western Blot and densitometry graphics corresponding to three independent experiments are showed (Mean ± SD). Load control: β-actin. Separate gels where used for cell line and tumour cells. ROD (Relative optic density). (**C**) mRNA levels resulting from RT-PCR analysis. Relative data to their corresponding P or P T population are presented in the graph. (*P ≤ 0.05; **P ≤ 0.01).

The expression and distribution of E-cadherin assessed by IF was variable (Fig. 6A). In ASZ, membrane localisation was only observed in P T cells, whereas the protein was weakly expressed in the cytoplasm of P, 10^th^G and 10^th^G T cells. In BSZ, while it was detected in both the cytoplasm and the membrane of P, 10^th^G and P T cells, cytoplasmic localisation was predominant in the 10^th^G and 10^th^G T populations being completely excluded from the membrane in the 10^th^G T cells. In CSZ P, P T and the polygonal cells of 10^th^G, E-cadherin was observed also in the cell membrane, while the spindled population did not express this protein.

The analysis by WB and RT-PCR (Fig. 6B,C) confirmed the results observed by IF; BSZ and CSZ 10^th^G showed lower expression of E-cadherin than their respective P populations; in ASZ cells, only the P T population showed expression of this protein.

**Figure 7:**
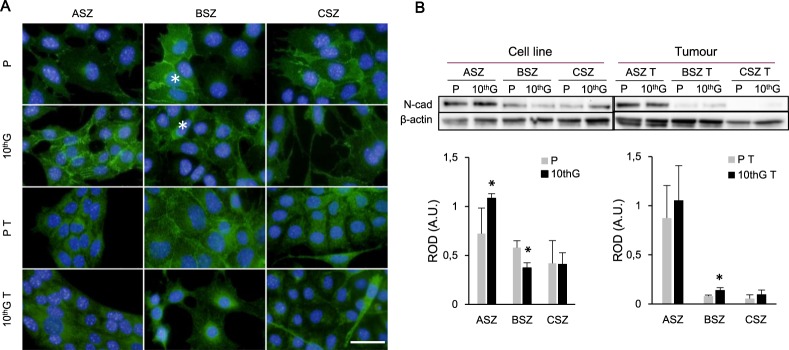
Localisation and expression of N-cadherin. (**A**) Immunofluorescence images of N-cadherin expression are shown (Green: N-cadherin; Blue: DNA stained with DAPI). In BSZ, cells with higher expression are indicated with an asterisk. Scale bar: 40 µm. (**B**) A representative experiment of N-cadherin expression by Western Blot and the densitometry graphics of three experiments are presented. Values are represented as mean ± SD (n = 3). Load control: β-actin. Separate gels where used for cell line and tumour cells. ROD: Relative optic density. (*P ≤ 0.05).

In the case of the EMT marker N-cadherin, in ASZ only 10^th^G cells showed expression of the protein in the membrane, while in ASZ P, P T and 10^th^G T cells the signal observed was diffuse in the cytoplasm. BSZ populations showed heterogeneous N-cadherin expression; some cells presented the protein in the membrane and in the cytoplasm, while others only in the cytoplasm. In CSZ P and 10^th^G N-cadherin was also presented in both, cytoplasm and membrane, but in P T and 10^th^G T cells it was only in the cytoplasm; spindled-shaped cells showed higher expression of the protein especially in 10^th^G T (Fig. 7A). The WB analysis revealed higher expression in ASZ 10^th^G and BSZ 10^th^G T cells related to their corresponding P. Conversely BSZ P presented higher expression than BSZ 10^th^G. No differences in the expression of N-cadherin were reported between CSZ populations (Fig. 7B).

Remaining focused on proteins implicated in EMT, we also evaluated the expression of vimentin. No differences in the expression or localisation of this protein were revealed either by IF or WB in any of the lines (Suppl. Fig. 4).

**Figure 8:**
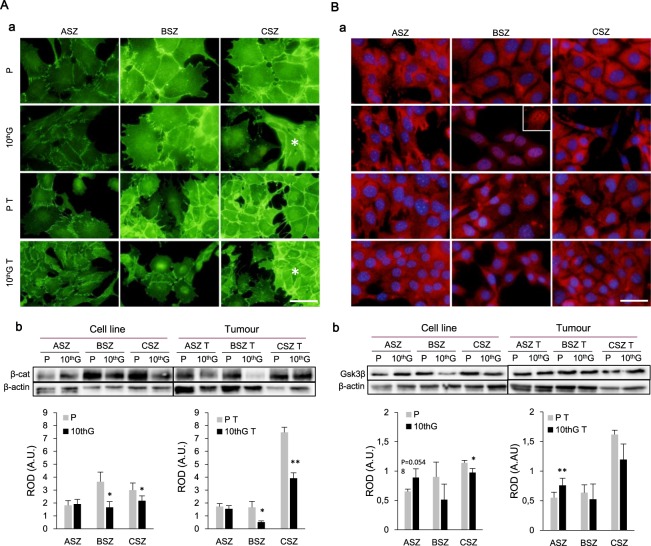
Localisation and expression of Wnt/β-catenin pathway components: β-catenin (**A**) and Gsk3β (**B**). (**A**a) Immunofluorescence images of β-catenin expression in green. In CSZ, polygonal population is indicated with an asterisk and spindled cells with and arrow. (**A**b) A representative experiment of β-catenin expression by Western Blot is shown, and the densitometry graphics. (**B**a**)** Immunofluorescence images of Gsk3β cellular localisation in red, and nuclei stained in blue (DAPI). In BSZ, a detail of the nucleus without DAPI is shown. (**B**b) A representative experiment of Gsk3β expression by Western Blot is shown, and the densitometry graphics (n = 3). Load control: β-actin. Separate gels where used for cell line and tumour cells. Separate gels where used for cell line and tumour cells. ROD: Relative optic density. Scale bar: 40 µm. (*P ≤ 0.05; **P ≤ 0.01). Values are represented as mean ± SD (n = 3).

Finally, we analysed potential variations in the expression of two important proteins of the Wnt/β-catenin pathway: β-catenin and Gsk3β. The IF assay revealed that β-catenin was located mainly in the membrane and diffusely in the cytoplasm, showing a more intense signal in BSZ and CSZ lines. Also, it was observed in the nucleus of some populations (ASZ P and P T, BSZ 10^th^G T). Spindled-shaped cells of CSZ 10^th^G and 10^th^G T populations presented lower β-catenin expression than polygonal cells (Fig. 8A). WB analysis confirmed these results; no changes were noticed for ASZ, while resistant populations of BSZ and CSZ decreased their expression of β-catenin.

In the case of Gsk3β, the protein was diffusely expressed in the cytoplasm of all lines except for BSZ 10^th^G, in which it was observed in the nucleus. In addition, the fluorescent signal was more intense in CSZ 10^th^G polygonal than in the spindled-shaped cells. WB analysis indicated higher expression in ASZ resistant (10^th^G and 10^th^G T) in relation to their parental cells. Conversely, in BSZ and CSZ resistant populations was lower than in the corresponding parental cells (Fig. 8B).

## Discussion

Several therapies are being used for the treatment of BCCs, including surgery, topical Imiquimod or PDT, and also specific inhibitors of the Hh signalling pathway (which is constitutively activated in most BCCs), such as the SMO inhibitor Vismodegib. However, in some cases, resistance of BCCs to clinical treatments occurs^21–27^. In the case of PDT for BCC, detection of recurrences after treatment has been as high as up to 30%^28–30^. Therefore, understanding the resistant mechanisms to PDT constitutes a very important goal for the management of BCC.

Several intrinsic and extrinsic factors have been described to be implicated in the resistance to PDT, including those related with the treatment –location and production of PS-, cancer cells features –proliferation, adhesion and expression of certain genes implicated on the development of the tumour including *p53* and components of *shh*/*ptch* and *Wnt/β-catenin* pathways^18,31^. In this study, we found that the resistance of BCC to PDT is associated to p53 expression, components of Wnt/β-catenin pathway and with the EMT process.

Cancer cells from different origin resistant to PDT *in vitro* have been obtained by using several PSs^15–17,32,33^. However, resistance of BCC cells to PDT has not been studied. In this context, we have used BCC cells (ASZ, BSZ and CSZ) isolated from mice with different genetic background^19^. BSZ and CSZ completely lack expression of p53, whereas ASZ expresses it. As previously described, in ASZ we determined mutations within the coding region of the gene at exon 5^20^. In any case the implication of such mutations in its function is unclear. From these cells we have obtained resistant cells to 10 cycles of MAL-PDT in order to stress the mechanisms of resistance.

We have detected changes in cell morphology. The 10^th^G populations of ASZ and BSZ appeared more spindled than their P. In CSZ, two populations were identified: one composed of polygonal cells, similar to their P, and the other one showing spindled-shaped cells that grew surrounding their polygonal counterparts. Changes to spindled shapes have also been seen in LM3 (mammary adenocarcinoma cells)^16^ and in SCC-13 cells^17^, resistant to PDT with ALA or MAL, respectively; in both cases, the authors related the morphological changes with increased aggressiveness. No differences were reported in the cell cycle distribution between P and 10^th^G cells in any of the lines, in agreement with previous studies in SCC-13^17^. However, size and complexity decreased in CSZ 10^th^G cells, as well as size in ASZ and BSZ 10^th^G. Conversely, it has been described an increase in size in RIF-1 murine fibrosarcoma cells resistant to Photofrin II-PDT^15,34^, and in LM3 resistant to ALA-PDT^16^; and in cancer cells resistant to chemotherapeutic agents^35,36^. In addition, lung cancer cells smaller in size appeared to be more sensitive to PDT with a benzoporphyrin derivative^37^. We consider that this lack of concordance between our results and those described by other authors, could be due to the different biology of BCC, an indolent and rarely metastatic tumour.

Proliferation analyses showed that ASZ (*p53*+) presented higher plating efficiency, generating more colonies, but of small size; while colonies of BSZ and CSZ (*p53−*) were larger, revealing higher proliferation ability. These results highlight the role of p53 in promoting adhesion to substrate and controlling proliferation^38,39^. No differences were seen in the total number of colonies between P and 10^th^G for any line, but we observed variable results related with the size in BSZ and CSZ: P cells formed a higher number of larger colonies than their corresponding 10^th^G. From the aforementioned results, it can be concluded that 10^th^G populations are less proliferative than their corresponding P but, in general, do not change neither the cell cycle dynamics nor the complexity.

Two relevant factors directly implicated in the efficacy of PDT are the production and the subcellular localisation of PpIX^9,16,31,40,41^. We have indicated no differences in PpIX production, between P and 10^th^G in ASZ and BSZ populations, supporting the results obtained in SCC-13 cells^17^. However, CSZ 10^th^G produced more PpIX than P after 24 h of MAL incubation, although the fluorescence signal was lower in the spindled than in the polygonal population; this difference could confer resistance to PDT in the former population. Supporting these results, primary murine keratinocytes treated with ALA accumulated more PpIX than Pam212 tumour cells^42^. In addition, the intracellular localisation has direct effects on the mode of cell damage^9,43^. Due to limited average life (40 s) and action radius (20 nm) of ^1^O_2_, the main ROS generated after MAL-PDT, different cell structures (e.g. mitochondria, lysosomes, plasmatic membrane and nucleus) in which PS is located represent primary targets^9,11,43,44^. Our results indicate that PpIX is localised on the cell membrane and also in the nucleus of ASZ and BSZ P cells, being related with a higher sensibility to PDT^45^ (as measured by MTT and AO-EB assay). Previous reports did not describe changes in localisation of PpIX between P and resistant RIF-1 cells -with ALA or Photofrin-^46^ nor SCC-13, localizing in the membrane^17^. In conclusion, the sensibility of P cells to PDT in ASZ and BSZ is favoured by nuclear PpIX localisation and in CSZ by its higher production.

Our results indicated that both P and 10^th^G cells were capable of generating tumours in mice after its inoculation with Matrigel. In addition, the tumours induced by 10^th^ G were bigger than those induced by P cells. This could point to a larger cancer stem cell component in the resistant populations^47^; although this should be confirmed. Similar results were obtained with the squamous cell carcinoma SCC-13^17,48^ and the mammary carcinoma 4T1^49^. Regarding to the type of tumours developed, no relevant histological differences were observed, except for the presence of osteoid tissue in the tumours generated from BSZ P, CSZ P and CSZ 10^th^G. These histological features presented similarities to one uncommon human BCC subtype, the basal cell carcinosarcoma, very rare in patients and with an equivalent metastasis capacity to conventional BCCs. However, we have not found conclusive differences between P and resistant cells^50^.

There are many evidences that connect resistance to anticancer therapies to the EMT process^51,52^. The EMT process is linked to morphological changes from epithelial/polygonal to fibroblastoid/spindled; phenotypical changes -loss of cell-cell adhesion mediated by E-cadherin- and an increase in the expression of mesenchymal markers, including vimentin or N-cadherin. It has been described that, among other factors, EMT can be promoted by the activation of Wnt/β-catenin pathway^53–55^. As mentioned before, the morphology of 10^th^G population of ASZ and BSZ underwent a slight elongation process and a new spindled-shaped cell population was observed in the cultures of CSZ 10^th^G. These changes could indicate an increased aggressiveness in the resistant BCC populations.

The best-studied indicator of EMT is the loss or decrease of E-cadherin expression, which confers a higher invasive capacity^56,57^. This was observed in the BCC resistant populations, except for the ASZ line. Lower expression of E-cadherin has been associated to more aggressive subtypes of BCC^58–60^. In the case of ASZ, neither P nor 10^th^G presented expression of E-cadherin, but it was expressed on the membrane and cytoplasm of P T cells, indicating a phenotypical change of ASZ P in the *in vivo* model.

Loss of E-cadherin is, in general, accompanied with expression of mesenchymal markers such as N-cadherin^61^. In this context, we did not find a total correlation between both proteins. The expression of N-cadherin was only increased in ASZ 10^th^G and BSZ 10^th^G T, and decreased in BSZ 10^th^G. Other authors have also related SCC progression with a decrease of E-cadherin expression but not with changes on N-cadherin expression^62^. Regarding vimentin, there are evidences indicating a relation between its expression and resistance to therapy depending on the tumours^63,64^. However, we have not found differences in its expression between the different populations, indicating that this protein may not be related with BCC resistance to PDT.

Since the EMT process is promoted by the activation of Wnt/β-catenin pathway, expression levels by WB and subcellular localisation of two proteins involved in this route were studied: β-catenin and Gsk3β kinase^65–67^. β-catenin was located in the membrane and cytoplasm of all ASZ populations and in ASZ P T was also detected in the nucleus. In resistant populations of BSZ and CSZ, its expression was lower than in P; and in BSZ 10^th^G cells was clearly also located in the nucleus. These data confirmed the lower adhesion of tumour resistant populations (10^th^G T) of the three cell lines. Besides, the loss of β-catenin expression in the membrane has been related to a bad prognosis in colorectal cancer^68^. In any case, these observations do not correlate to those described in SCC-13 cells, where no differences in E-cadherin and β-catenin expression patterns were reported^69^. Even so, Casas *et al*.^70,71^ described the deregulation of E-cadherin/β-catenin complex in LM3 resistant cells, but no decrease in protein expression levels was found. Regarding Gsk3β, its expression has been reported to be decreased in cells with constitutive activation of the pathway, as it occurs in most solid tumours^72,73^. In this study, we show that Gsk3β expression increased in resistant populations of ASZ and decreased in those of BSZ and CSZ (particularly in the spindled cells). Moreover, Gsk3β was located in the nucleus of BSZ 10^th^G cells, which has been related to apoptosis inhibition^74^ and replicative senescence induction^75^. The role of this protein in the resistance processes is not well defined. Whereas an increase in its expression has been linked to gemcitabine and radiation resistance in pancreatic cancer cells^76^, decreased expression has been reported in lung adenocarcinoma cells resistant to cisplatin^77^.

All these observations, together with the evidences that relate the lack of *p53* with the increase in expression of components of the Wnt/β-catenin pathway, which favour EMT^38,78–81^, allow us to hypothesize two mechanisms of BCC resistance to MAL-PDT.

BSZ and CSZ cell lines lack expression of p53 whereas ASZ cell line expresses the gene at the RNA and the protein level. Therefore, in the case of ASZ, in which point mutations have been described, it is possible that (1) the gene in not completely inactivated by the mutations and retains some wt function, or (2) the mutations confer advantages in promoting tumorigenesis. In relation to (1), whereas the majority of tumour suppressor genes (RB, APC or BRCA) are inactivated during cancer progression, by mutations, the *p53* gene is often found to undergo missense mutations. In relation to (2), there is growing evidence that mutant p53 have lost wt p53 tumour suppressor activity and gained functions that contribute to malignant progression^82^. In addition, *in vivo* experiments showed that mice expressing mutant p53 display a tumour profile that is more aggressive and metastatic than p53 null or wild-type mice^83^. From the results obtained, we cannot indicate what occurs in ASZ, further studies must be done. In any case, the results indicate that resistant ASZ cells, exhibited differential characteristics to those observed in BSZ and CSZ. *p53* expression (ASZ) favours the decreased expression of β-catenin and increased that of Gsk3β, repressing Wnt/β-catenin pathway, especially in resistant populations. Cell adhesion is lower in this line and the EMT process would be associated with an increase in N-cadherin expression in resistant populations. In the absence of expression of *p53* (BSZ and CSZ), Gsk3β levels decrease and nuclear β-catenin becomes more evident in resistant cells, which seems to indicate a higher activity of the Wnt/β-catenin pathway. The EMT process, in this case, is associated to decreased adhesion (lower expression of E-cadherin and β-catenin in the membrane), but not with higher expression of mesenchymal proteins such as N-cadherin, except for the BSZ 10^th^G, in which this protein is increased in relation to P cells.

## Materials and Methods

### Cell lines and culture

The study has been performed by using cell lines (kindly provided by Dr. Epstein’s laboratory) obtained from BCCs induced in a *ptch1*^+/−^ mouse by UV irradiation (ASZ001, ASZ); in a *ptch1*+/−, K14CreER2/+; p53fl/fl mouse exposed to γ radiation (BSZ2, BSZ) and from a spontaneous tumour developed in a *ptch1*+/−, K5-CrePR, p53fl/fl mouse (CSZ1, CSZ)^19,20^. All cell lines were cultured in DMEM (Thermo Scientific, Hyclone) supplemented with 10% foetal bovine serum (FBS) (Thermo Scientific, Hyclone) and 1% of Penicillin/Streptomycin (Thermo Scientific, Hyclone). The cells were grown at 37 °C in an atmosphere with 5% of CO_2_.

### PDT and generation of resistant populations

The procedure followed for obtaining PDT resistant populations was based in those previously published^15–18^. For PDT, MAL (Sigma) was used as a precursor of the endogenous PS PpIX. Cells seeded in F12.5 were incubated for 5 h with different concentrations of MAL in FBS-free DMEM and thereafter subjected to red light irradiation. The light source employed was a red LEDs lamp (635 nm) (Segainvex, UAM). Treatment conditions that caused survival rates of 5–15% were chosen (tested by MTT assay). The selected initial treatment conditions that induced a survival rate lower than 15% were: 0.3 mM, 0.4 mM or 0.2 mM MAL for ASZ, BSZ or CSZ, respectively and a light dose of 2 J/cm^2^. For the rest of the PDT cycles, the concentration of MAL was maintained and light doses were increased if necessary, to obtain surveillance between 5–15%. After each cycle, surviving cells were allowed to reach 50–60% of confluence before applying a new PDT treatment^15,17^. The final population received a total of 10 cycles of PDT. The initial population, not subjected to PDT, was called parental population (P), and 10^th^G refers to the population resistant to 10 cycles of PDT. For the experiments, P and 10^th^G cells were used until 7 passages.

### Cytotoxic assays

The evaluation of the cytotoxic effect of PDT was performed by the MTT assay (Sigma). Cell viability was tested 24 h after each PDT treatment by incubating the cultures with 500 µg/mL of a MTT solution for 3 h. Formazan crystals were dissolved in DMSO (Panreac) and the optic densitometry was measured in a Spectra Fluor, Tecan reader plate at 542 nm. Cells with neither drug nor light exposition were used as controls and their viability rate was expressed as 100%. From the survival values obtained from a determined irradiation conditions (1.36 or 2.36 J/cm^2^) fold-change indexes were calculated as the ratio between the % of survival of each 10^th^G and P cell type (Fold-change = %10^th^G survival/%P survival). The experiments were performed, at least, three times.

Cell death was also evaluated by the acridine orange (AO, Sigma) and ethidium bromide (EB, Sigma) assay that allowed distinguishing between viable and dead cells. After 24 h of MAL-PDT, AO and EB were added to the cultures at a final concentration of 50 µg/ml. Immediately after staining the cells were observed in the fluorescence microscope under blue excitation light (450–490 nm, BP 490). According to the fluorescence colour observed, cells were classified as viable and dead fluorescing in green or orange, respectively.

### Tumorigenic assay and Isolation of BCC keratinocytes

For the tumorigenic assays, 8 week-old *Athymic Nude-Foxn1*^*nu*^ mice (Envigo, France) were used. Mice were classified randomly in 3 groups with 5 mice per group. Each mouse was inoculated in the left flank with 1.5 × 10^6^ of P cells in 50 µL of PBS and 50 µL of Matrigel (Corning), and in the right flank with the same number of 10^th^G cells. During the subsequent days, the animals were monitored, measuring the progressive increase of tumour size with an automatic calliper. The sum of all the different lobules was considered the tumour volume, and it was calculated with the following formula:

$$V=\frac{4}{3}\pi \,\ast \,{(\frac{\frac{lenght+with+depth}{3}}{2})}^{3}$$

When the tumour reached the maximum established volume of 500 mm^3^, mice were sacrificed with CO_2_. Tumours were surgically removed and divided into 2 pieces, one for histopathological evaluation and the second one for tumour keratinocytes isolation. In the first case, pieces were fixed with 3.7% formaldehyde (Panreac) in PBS (Thermo Scientific, Hyclone), washed in PBS and included in paraffin. In the second case, tumour cell cultures were performed by explant. For this end, the tissue was washed with 96% ethanol (Panreac), then in ethanol/PBS 1:1 and three times with PBS in sterile conditions. After that, the tumours were mechanically disaggregated and seeded on 35 mm Petri plates with DMEM supplemented with 1% P/S, 10% FBS and 1% amphotericin B. When keratinocyte colonies started to form, they were isolated by trypsinization and amplified. Cells obtained from tumours induced by P or 10^th^G cells were named P T or 10^th^G T, respectively^84^.

All methods were carried out in accordance with relevant guidelines and Spanish regulations. All experimental protocols were approved by the Committee of ethical investigation of Autonomous University (CEI-85-15809) and the Committee of ethic in human and animal experimentation of CSIC (Centro Superior de Investigaciones Científicas) on the 15^th^ of June 2015 (number 280790000188).

### Cell morphology and immunofluorescence

To analyse the cell morphology, cells were cultured over coverslips and observed on a phase contrast microscopy connected to a CCD DP70 camera (Olympus BX-61).

For immunofluorescence (IF), cells grown on coverslips were fixed in 3.7% formaldehyde/PBS, permeabilized with 0.1% Triton X-100/PBS (20 min RT) and incubated for 1 h at 37 °C with the primary antibodies anti-E-cadherin, β-catenin, N-cadherin and Gsk3β (Suppl. Table 1). Then, cells were washed in PBS and incubated with secondary antibodies (Suppl. Table 1). Finally, cells were washed in PBS and mounted in ProLong-DAPI (Invitrogen). Images were taken with an epifluorescence microscope linked to an Olympus DP50 digital camera and using the following excitation filters for fluorescence: UVA (365–390 nm, UG-1) for DAPI, blue (450–490 nm, BP 490) for Alexa Fluor 488 and green (510–550 nm, DM 6590) for Alexa Fluor 546 dyes.

### Size, complexity and cell cycle

Size and complexity parameters and cell cycle distribution were analysed by flow cytometry (Cytomics FC500, 1 laser, Beckman Coulter). For that, cells were trypsinized and washed with PBS by centrifugation. Size evaluation was made based on forward scatter and the complexity was evaluated by side scatter. For cell cycle analysis, DNAprep kit (Beckman Coulter) was used. The pellet obtained by centrifugation was resuspended in 50 µL of detergent of the kit, and 1 mL of propidium iodide, incubating 30 min at 37 °C. Cells were maintained at 4 °C in dark until performing the measurement.

### Cell proliferation

Proliferation rate was estimated using the clonogenic assay. Cells were seeded at 125 cells/mL and they were let to grow for 7 days. Then, cultures were washed with cold PBS (4 °C) and stained with 0.2% crystal violet (Aldrich Company, Inc) in 2% ethanol in distilled water for 20 min in constant shaking at room temperature. Thereafter, the cultures were washed with PBS, plates were let to dry and colonies (each colony is formed by >50 cells) were counted in number and classified in groups by their size as: small (<1.5 mm), medium (>1.5 mm; <2.5 mm) and big (>2.5 mm). Next, cells were lysed and dye was dissolved in 1% SDS (Sodium Dodecyl Sulfate) (Panreac); and optic density was measured with a plate reader at 570 nm (Spectrafluor, Tecan).

### Production and subcellular localisation of PpIX

The production of PpIX was evaluated by flow cytometry (FC500 Cytomics 2 lasers, Beckman Coulter) after incubation with the appropriate concentrations of MAL for 5 or 24 h. After MAL incubation cells were trypsinized, centrifuged 7 min at 481 g and fixed with 1 mL of 3.7% formaldehyde in PBS at room temperature for 10 min. Fixing solution was removed washing the cells with PBS twice by centrifugation, resuspended in clean PBS and kept in the dark at 4 °C until evaluation. PpIX emission measurements were obtained employing the flow cytometer Cytomics FC500 (λexc = 625 nm; λem = 670 nm). Fluorescence intensity was determined for 10^4^ cells per each cellular population. PpIX subcellular localisation was determined by fluorescence microscopy using the UVA excitation line. For that, cells were seeded on cover slips and incubated or 24 h at 37 °C with different concentrations of MAL in DMEM supplemented with 1% FBS. Then, cells were briefly washed with PBS, mounted on slides and observed *in situ* under the fluorescence microscope using UVA excitation light; PpIX appeared fluorescing in red.

### RNA and DNA extraction and PCR

The mRNA was isolated using a mini RNeasy kit (Qiagen). RNA concentration and purity was determined by spectrophotometry (NanoDropND1000, Nanodrop Technologies). Expression of mRNA was evaluated by RT-PCR using the corresponding reagents and specific primers: *E-cadherin* (5′ATCCTCGCCCTGCTGATT 3′; 3′ ACCACCGTTCTCCTCCGTA 5′), *p53 wt* (5′ GCAACTATGGCTTCCACCTG 3′; 3′ TTATTGAGGGGAGGAGAGTACG 5′), *ptch wt* (5′ CTGCGGCAAGTTTTTGGTTG 3′; 3′ AGGGCTTCTCGTTGGCTACAAG 5′) and *mutated ptch* (5′ GCCCTGATGAACTGCAGGACG 3′; 3′ CACCGGGTAGCCAACGCTATGTC 5′). The results were analysed using the DDt method (software LC480 1.5) that adjusts Cq data of each sample in relation to the value of the reference gene (18S). As a control of *ptch* wildtype expression 3T3 cells (ATCC) were employed.

ASZ was also screened for p53 point mutations by PCR amplification and sequenced by Sanger Method. For that, DNA was isolated from cultures by using DNeasy Blood & Tissue Kit (Qiagen). Primers were design with Perlprimer program to evaluate exon 5 and exon 8 regions. Genomic sequence was obtained from the National Center for Biotechnology Information (NCBI) Gene database (Accession number NC_000077.6). Primers for exons 5 were (5′ GCCTGGTCTACAAAGTGAGTTCC 3′; 3′ CACCCGGATAAGATGCTGGG 5′), and for exon 8 were (5′GCGTGGTAGGTTAGGTTAGC 3′; ´GTGAAATACTCTCCATCAAGTGGT5′). The amplification products were purified with Monarch PCR & DNA Cleanup Kit (NEB) following the manufacturer’s recommendations. Fragments were sequenced in a capillary electrophoresis instrument AB3730XL (Applied Biosystems). Specific codons were analysed for exon 5 (137, 176 and 149) and for exon 8 (275) according to previously published data^20^.

### Western Blots

Protein extracts were obtained using RIPA buffer/Triton pH 7.4 (Bioworld) with inhibitors of phosphatases (PhosSTOP EASYpack, Roche) and proteases (cOmplete ULTRA tablets Mini EDTA-free EASYpack, Roche), following commercial brand indications. Protein concentration was determined by BCA Protein Assay Kit (Thermo Scientific Pierce). Subsequently, extracts were diluted in Laemmli buffer (Bio-Rad), heated for 5 min at 98 °C and electrophoresis was performed in acrylamide/bisacrylamide gels in denaturing conditions (SDS-PAGE), using a Miniprotean base, and Western Blotting by Transblot Turbo system (Bio-Rad) to mmobilon-P PVDF membrane (Bio-Rad). Membranes were incubated with blocking solution of skimmed milk 5% in TBS with 0.1% Tween20, for at least one hour at room temperature shaking. Afterwards, the membranes were incubated with primary antibodies overnight at 4 °C shaking (Suppl. Table 1), washed with TBS-T and incubated with a secondary antibody conjugated with peroxidase (Suppl. Table 1) for 2 h at room temperature. Quimioluminiscence revealing (kit ECL Plus, Amersham) was carried out using the high resolution system ChemiDocTR XRS+ (Bio-Rad). Bands were digitalized using Image Lab version 3.0.1 (Bio-Rad) software.

### Histology

Mouse tumour samples fixed in 3.7% formaldehyde in PBS for 12 h at 4 °C were included in paraffin. Histologic sections (5 µm) were mounted on slides with electrostatic charge (Superfrost Plus, Thermo Fisher). Sections were then deparaffinised (15 min in xilol), and stained with haematoxylin/eosin (H/E), for general structure, or alizarin red, for calcium deposits. For the first one, sections were stained with haematoxylin for 5 min, and then with eosin for 6 min (Panreac). For alizarin red staining, sections were immersed in 0.1% of colorant in 0.5% KOH in distilled water, pH 4.1–4.3, dried with acetone, acetone/xilol 1:1 and xilol. Finally all sections were mounted with DepeX.

### Statistical analysis

The *in vitro* experiments were repeated at least three times. ANOVA and t-test were used, in general, to determinate statistical differences. For tumour volume and number of colonies, a nonparametric Mann-Whitney U test (*P* ≤ 0.05) was used; and the Kruskall-Wallis test was used to compare three or more populations. Data are expressed as the mean value ± standard deviation (SD) or standard error (SE).

## Supplementary information

Supplementary information

## Data Availability

The datasets generated during and/or analysed during the current study are available from the corresponding author on reasonable request.
