# Supplementary material for: Characterisation of resistance mechanisms developed by basal cell carcinoma cells in response to repeated cycles of Photodynamic Therapy
Source: Sci Rep. 2019 Mar 18;9:4835. doi: 10.1038/s41598-019-41313-y (PMC6423284; doi:10.1038/s41598-019-41313-y)
Supplement: Supplementary file 1 — Supplementary information [file 41598_2019_41313_MOESM1_ESM.pdf]

“Characterisation of resistance mechanisms developed by basal cell carcinoma cells in response to repeated cycles of Photodynamic Therapy”

Lucena SR[1], Zamarrón A[1], Carrasco E[2], Marigil MA[3], Mascaraque M[1], Fernández-Guarino M[4], Gilaberte Y[5], González S[6], Juarranz Á[17].

<u>Supplementary Table 1:</u> Primary and secondary antibodies used for immufluorescence (IF) and Western Blot (WB) analyses.

Antibody	*Manufacturer*	*Method*
Primary		
Mouse monoclonal anti-E-cadherin	BD Transduction Laboratories	WB, IF
Mouse monoclonal anti-N-cadherin	Zymed	WB, IF
Mouse monoclonal anti-β-actin	Abcam	WB
Mouse monoclonal anti-β-catenin	BD Transduction Laboratories	WB,IF
Rabbit monoclonal anti-gsk3β	Abcam	WB, IF
Rabbit polyclonal anti-p53	Novocastra	WB
Rabbit polyclonal anti-vimentin	Abcam	WB,IF
Secondary		
Goat anti-mouse Alexa Fluor® 488	Life Technologies	IF
Goat anti-mouse IgG peroxidase conjugate	Thermo Fisher	WB
Goat anti-rabbit Alexa Fluor® 546	Invitrogen	IF
Goat anti-rabbit IgG Horseradish peroxidase conjugate	Thermo Fisher	WB

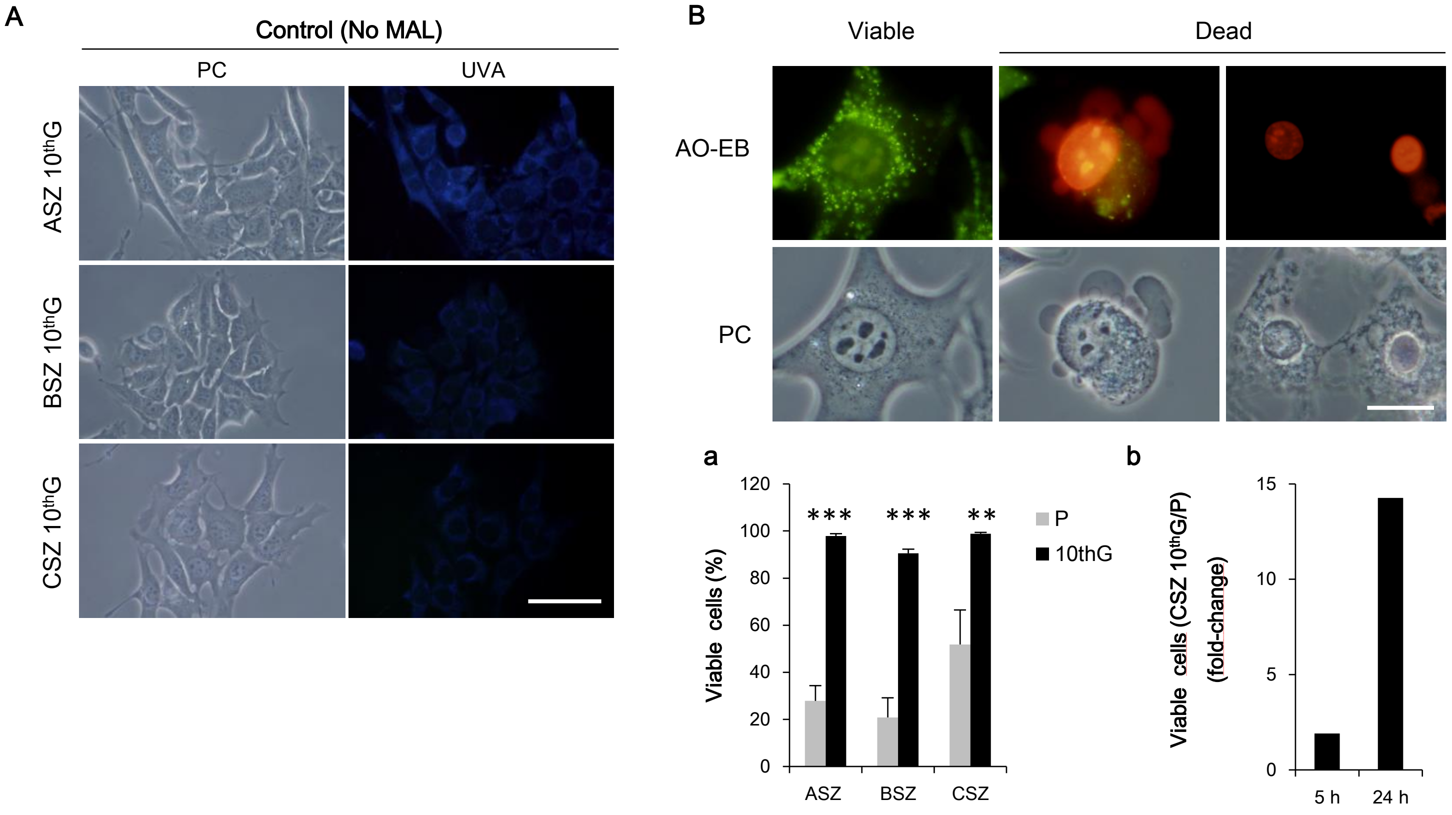

<u>**Supplementary Figure 1:**</u> **Control cells under UVA excitation light and live/dead assay after PDT treatment.** (**A**) Images of mitochondria's autofluorescence (blue) in 10thG cells without MAL incubation (Control) under UVA light excitation. (**Ba**) Acridine orange (AO) (orange)-Ethidium bromide (EB) (green) assay was performed in P and 10thG cells after 5 h of MAL incubation (0.3 mM ASZ, 0.4 mM BSZ, 0.2 mM CSZ) and red light irradiation. Values are represented as mean ± SD (n=3) (**: $P \leq 0.01$; ***: $P \leq 0.001$). (**Bb**) In CSZ P and 10thG cells same experiment as in (Ba) was performed after 24 h of incubation with MAL. Fold-change (% viable cells of 10thG / % viable cells of P) is represented for 5 and 24 h of incubation with MAL.

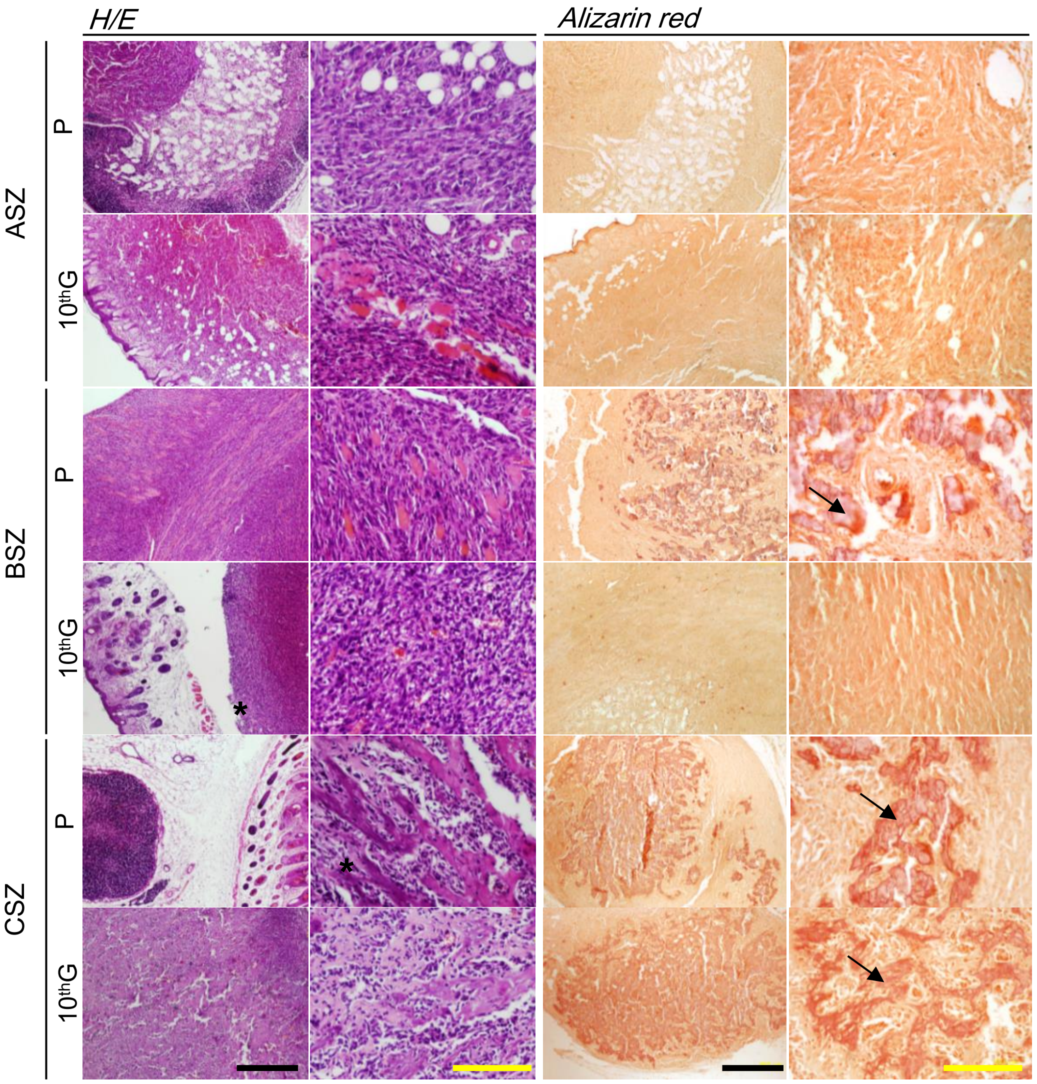

<u>Supplementary Figure 2:</u> Histological analysis of murine tumours generated by ASZ, BSZ and CSZ (P and 10thG) inoculation. A representative image of Haematoxylin/Eosin and alizarin red staining of each type of tumour is showed at two different scales. Arrows indicate the presence of osteoid. All the images are focused on areas of skin affected by BCC. Asterisks indicate surrounding healthy skin remaining unaffected. Scale bar for all the images of each column (black: 500 µm or yellow: 150 µm) is show on the bottom panel.

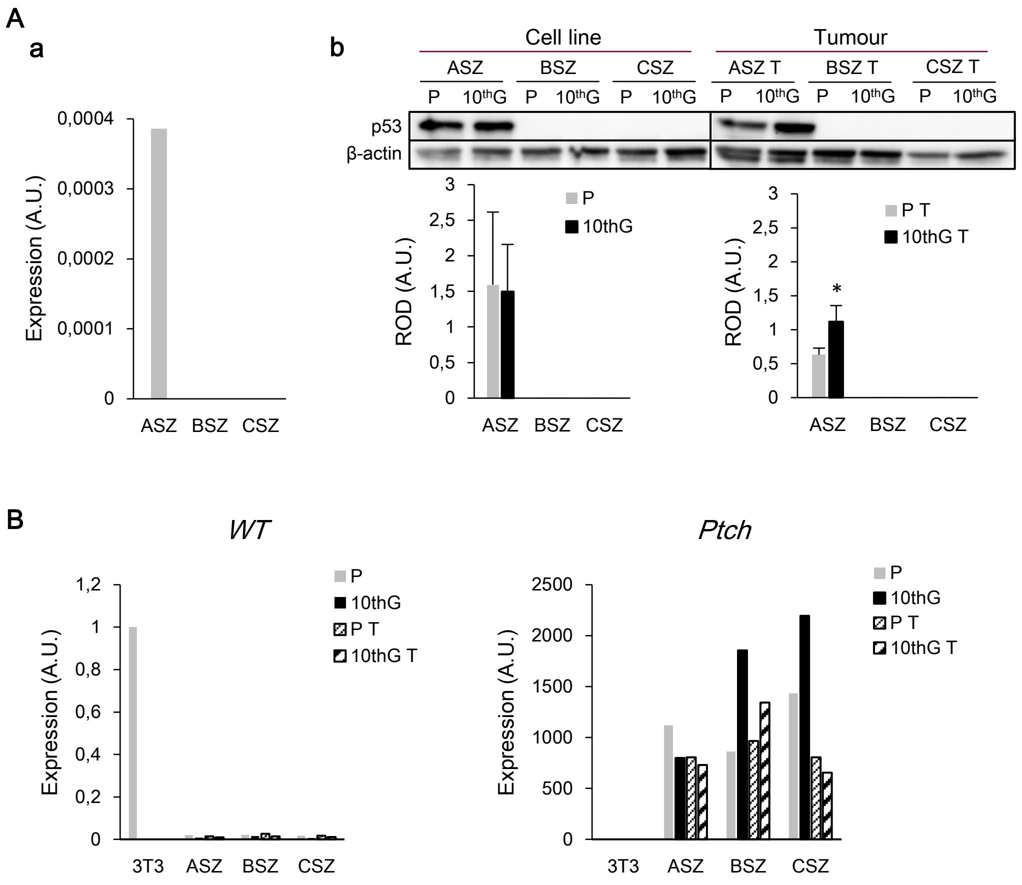

<u>Supplementary Figure 3:</u> Expression of *p53* and *ptch*. (**Aa**) *p53* expression in P populations by RT-PCR. (**Ab**) p53 expression in all populations analysed by Western Blot. β-actin expression is used as load control. Separate gels where used for cell line and tumour cells. ROD: Relative optic density Values are represented as mean ± SD (n=3). (**B**) The expression of *ptch* was analysed by RT-PCR. All data are related to the expression of the *WT* or mutated (*Ptch*) alele in 3T3 cells. (*:*P*≤0.05).

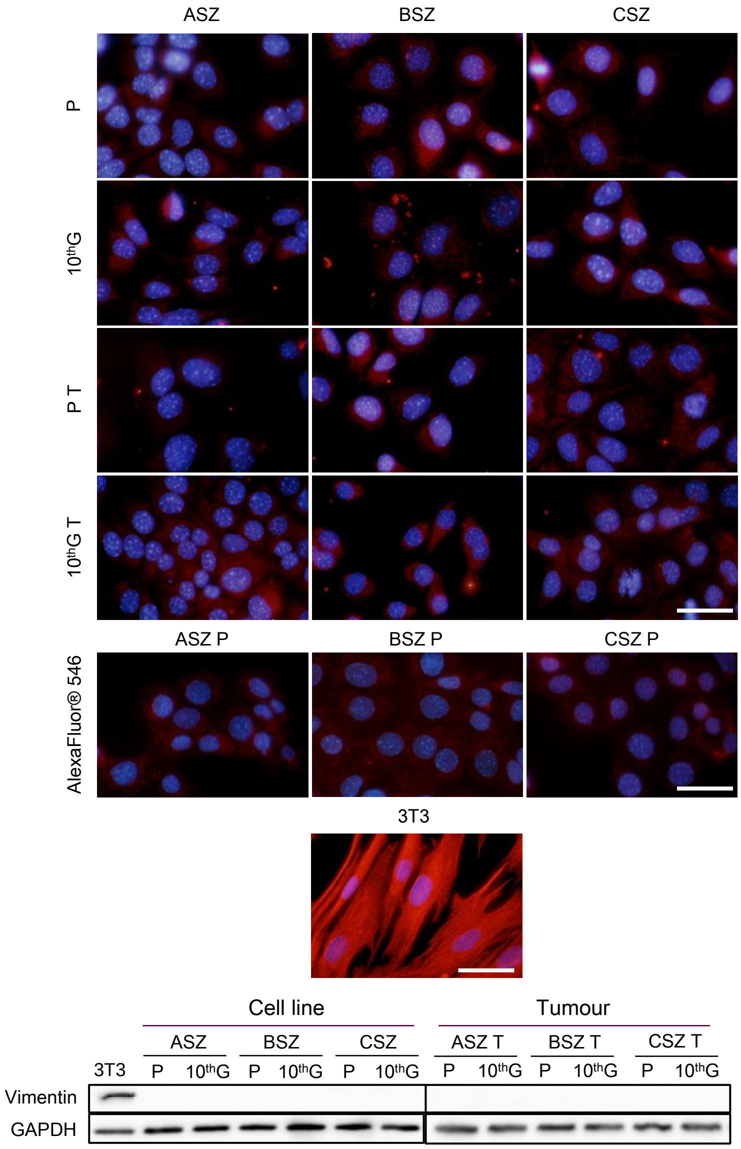

<u>Supplementary Figure 4:</u> Localisation and expression of vimentin. Fluorescence microscopy images (Red: vimentin; Blue: DNA stained with DAPI) and Western Blot for the detection of vimentin are presented. 3T3 cells were used as a positive control for vimentin expression assessed by IF and WB. Scale bar: 40µm. A secondary antibody control (AlexaFluor 546) is included in P cells. GAPDH was used as load control. Separate gels where used for cell line and tumour cells but performed in same conditions at the same time.
